# Phosphorylation of RyR2 Ser‐2814 by CaMKII mediates β1‐adrenergic stress induced Ca^2+^‐leak from the sarcoplasmic reticulum

**DOI:** 10.1002/2211-5463.13274

**Published:** 2021-09-02

**Authors:** Maria J. Baier, Jannis Noack, Mark Tilmann Seitz, Lars S. Maier, Stefan Neef

**Affiliations:** ^1^ Department of Internal Medicine II University Medical Center Regensburg Germany; ^2^ Department of Trauma Surgery, Orthopaedics and Plastic Surgery University Medical Center Göttingen Germany

**Keywords:** adrenergic stress, CaMKII, RyR2, Ser‐2814, SR Ca^2+^ leak

## Abstract

Adrenergic stimulation, while being the central mechanism of cardiac positive inotropy, is a universally acknowledged inductor of undesirable sarcoplasmic reticulum (SR) Ca^2+^ leak. However, the exact mechanisms for this remained unspecified so far. This study shows that Ca^2+^/calmodulin‐dependent protein kinase II (CaMKII)‐specific phosphorylation of ryanodine receptor type 2 at Ser‐2814 is the pivotal mechanism by which SR Ca^2+^ leak develops downstream of β1‐adrenergic stress by increase of the leak/load relationship. Cardiomyocytes with a Ser‐2814 phosphoresistant mutation (S2814A) were protected from isoproterenol‐induced SR Ca^2+^ leak and consequently displayed improved postrest potentiation of systolic Ca^2+^ release under adrenergic stress compared to littermate wild‐type cells.

## Graphical abstract

This study shows that Ca^2+^/calmodulin‐dependent protein kinase II‐specific phosphorylation of ryanodine receptor type 2 at Ser‐2814 is the pivotal mechanism by which sarcoplasmic reticulum Ca^2+^ leak develops downstream of β1‐adrenergic stress by increase of the leak/load relationship.

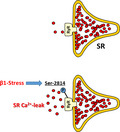

**Abbreviations**
A/Ala: alanine
CaMKII: Ca^2+^/calmodulin‐dependent protein kinase II
ISO: isoproterenol
NT: normal Tyrode’s solution
PKA: protein kinase A
PLB: phospholamban
RyR2: ryanodine receptor type 2
S/Ser: serine
SERCA2a: sarcoplasmic reticulum Ca^2+^ ATPase type 2a
SR: sarcoplasmic reticulum
WT: wild‐type

Adrenergic activation (the ‘fight or flight’ response) is the central mechanism by which the cardiovascular system responds to stress, resulting in acute positive inotropy due to β1‐adrenergic activation. However, β1‐adrenergic stimulation is also a strong and universally acknowledged inductor of sarcoplasmic reticulum (SR) Ca^2+^ leak [1]. And while β1‐adrenergic stimulation acutely has positive inotropic effects and can be acutely necessary for cardiac decompensation/shock, it is arrhythmogenic and chronic adrenergic stress is even detrimental to cardiac function. It is believed that SR Ca^2+^ leak plays a central role in these detrimental consequences of adrenergic stimulation [2, 3].

The molecular mechanisms by which Ca^2+^ leak from the SR acutely or chronically develops in the heart are complex and have been subject to controversy and discrepant findings. Activation of the protein kinase A (PKA) pathway downstream of β1‐adrenergic receptor stimulation increases SR Ca^2+^ uptake via disinhibition of SR Ca^2+^ ATPase type 2a (SERCA2a) due to phospholamban (PLB) phosphorylation by PKA [4] and increased SR Ca^2+^ loading has been shown to facilitate Ca^2+^ loss from the SR (‘leak/load relationship’) [5]. This relationship between increased SR Ca^2+^ content and induction of SR Ca^2+^ leak is exponential [6], with extremely increased SR Ca^2+^ content leading to a phenomenon called ‘spillover’ [7]. Another important mechanism for the (patho)genesis of SR Ca^2+^ leak is increased open probability of the SR Ca^2+^‐release channel ryanodine receptor type 2 (RyR2) due to increased phosphorylation of this protein [8, 9]. The RyR2 has multiple phosphorylation sites, the major ones being Ser‐2030, Ser‐2808, and Ser‐2814. Both phosphorylation of the RyR2 by PKA (at Ser‐2808) and by Ca^2+^/calmodulin‐dependent protein kinase II (CaMKII, at Ser‐2814 and possibly also Ser‐2808) have been suggested to be important for pathologic RyR2 gating [10, 11]. This is important also in cardiac disease, where, for example in heart failure and atrial fibrillation, increased SR Ca^2+^ leak has been attributed to RyR2 hyperphosphorylation and appears to play a role in the pathophysiology of both conditions [12, 13]. In addition, it was shown that mice with a constitutively unphosphorylatable Ser‐2814 phosphorylation site (Ser2814Ala mutation, S2814A mice, [14]) were protected from pressure overload‐induced heart failure [12].

An important insight into the development of SR Ca^2+^ leak induced by adrenergic stress came from Curran *et al*. [15], who reported that, surprisingly, not PKA but CaMKII is the essential downstream mechanism for SR Ca^2+^ leak upon β1‐adrenergic receptor stimulation with isoproterenol (ISO). However, CaMKII is broadly involved in the (dys)regulation of cardiomyocyte Ca^2+^ handling and the exact mechanisms by which CaMKII mediates the β‐adrenergically induced SR Ca^2+^ leak remained unspecified so far. Possible mediators include increased CaMKII‐dependent phosphorylation of RyR2, increased SR Ca^2+^ loading (via leak/load relationship) due to CaMKII‐mediated increased L‐type Ca^2+^ current influx (upon L‐type Ca^2+^ channel phosphorylation by CaMKII), or enhanced SR Ca^2+^ load as a consequence of CaMKII‐dependent phosphorylation of PLB (at Thr‐17, thus disinhibiting SERCA2a and increasing SR Ca^2+^ uptake).

Importantly, SR Ca^2+^ leak is comprised of small fraction of Ca^2+^‐spark‐mediated and a (quantitatively clearly dominant) nonspark fraction [16, 17, 18]. Thus, we used tetracaine‐sensitive Ca^2+^ shift [5] to assess SR Ca^2+^ leak, as this measures global SR Ca^2+^ leak (nonspark plus spark‐mediated leak) [19].

## Materials and methods

### Animals

S2814A mice and the respective wild‐type (WT) littermates were a friendly gift from Prof. Stephan Lehnart (Göttingen, Germany) and X. Wehrens (Houston, TX, USA), backcrossed to a C57N background at the central animal research facility of the University Medical Center Regensburg. Animals were housed at a 12‐h light–dark cycle in cages type IIL. Standard laboratory chow and water were provided ad libitum. Heterozygous breeding resulted in WT and S2814A littermates that were used for the experiments (male and female mice, 12–16 weeks). The investigation conforms to the Guide for the Care and Use of Laboratory Animals published by the US National Institutes of Health (NIH Publication No. 85‐23, revised 1985). All institutional and national guidelines for the care and use of laboratory animals were followed and approved by the appropriate institutional committees.

### Isolation of cardiomyocytes

Isolation of cardiomyocytes was performed as described previously [20]. Briefly, mice were anesthetized with isoflurane, and hearts were quickly excised. After weighing, hearts were mounted on a Langendorff perfusion apparatus and retrogradely perfused with nominally Ca^2+^‐free solution containing (in mmol·L^−1^): 113 NaCl, 4.7 KCl, 0.6 KH_2_PO_4_, 0.6 Na_2_HPO_4_, 1.2 MgSO_4_, 12 NaHCO_3_, 10 KHCO_3_, 10 HEPES, 30 taurine, 10 BDM (2,3 butanedione monoxime), 5.5 glucose, 0.032 phenol‐red for 4 min (37 °C, pH 7.4). Then, 7.5 g·L^−1^ liberase™ (Roche diagnostics, Mannheim, Germany), trypsin 0.6%, and 0.125 mmol·L^−1^ CaCl_2_ were added to the perfusion solution and perfusion was continued until the heart became flaccid. Ventricular tissue was collected in perfusion buffer containing 5% bovine calf serum, cut into small pieces, dispersed, and filtered, until no solid tissue was left. After Ca^2+^ reintroduction by stepwise increasing [Ca^2+^] from 0.1 to 1.4 mmol·L^−1^, cardiomyocytes were plated onto superfusion chambers, which had been coated with laminin to allow cell adhesion.

### Intracellular Ca^2+^ measurements

Intracellular Ca^2+^ measurements were performed as described previously [20] using an epifluorescence detection system (IonOptix Corp, Milton, MA, USA) mounted to a Nikon TE2000U inverted microscope. Isolated cells were loaded with Fluo‐4 AM (10 µmol·L^−1^) for 15 min and superfused with normal Tyrode's solution (‘NT’) consisting of (in mmol·L^−1^): 140 NaCl, 4 KCl, 5 HEPES, 1 MgCl_2_, 10 glucose, 2 CaCl_2_ (pH 7.4 with NaOH). Excitation of Fluo‐4 was at 480 ± 15 nm, emission was collected at 535 ± 20 nm. Myocytes were field‐stimulated at 1 Hz until steady‐state was achieved. For β1‐adrenergic stimulation, ISO (100 nmol·L^−1^) was added to the superfusion (starting for 5 min before recording first data). Sarcoplasmic reticulum (SR) Ca^2+^ content was estimated by rapid application of caffeine (10 mmol·L^−1^).

### SR Ca^2+^ leak measurements using tetracaine

Tetracaine experiments to measure SR Ca^2+^ leak were performed according to the method of Shannon *et al*. [5]. Na^+^‐ and Ca^2+^‐free bath solution (‘0Na^+^/0Ca^2+^’) was prepared consisting of (in mmol·L^−1^): 140 LiCl, 4 KCl, 5 HEPES, 1 MgCl_2_, 10 glucose, 10 EGTA, 2 CaCl_2_ (pH 7.4 with LiOH). Tetracaine 1 mmol·L^−1^ was added to this solution to prepare the tetracaine solution. The fractional shift in diastolic fluorescence upon tetracaine (an allosteric blocker of ryanodine receptors) under 0Na^+^/0Ca^2+^ conditions was measured after rapid switching of superfusion solutions using local application immediately at the cell, followed by caffeine application (10 mmol·L^−1^) to calculate leak/load relationship.

### Western blot

After isolation of S2814A and WT cardiomyocytes (see above), cells were either incubated in NT or ISO solution (100 nmol·L^−1^) for 10 min. Cells were homogenized in Tris buffer containing (in mm): 20 Tris/HCl, 200 NaCl, 20 NaF, 1 Na_3_VO_4_, 1 DTT, 1% Triton X‐100 (pH 7.4), complete protease inhibitor cocktail (Roche diagnostics, Mannheim, Germany) and phosphatase‐inhibitor mixture (PhosSTOP; Roche). Protein concentration was determined by BCA assay (Sigma‐Aldrich Co., St. Louis, MO, USA). Denatured proteins were separated on SDS‐polyacrylamide gels (5–12%) and transferred to nitrocellulose membranes (GE Health Care, Chalfont St Giles, UK). Specific proteins were detected using anti‐RyR2 (1 : 10 000, Sigma‐Aldrich Co.), anti‐pS2814‐RyR2 (1 : 1000; Badrilla, Leeds, UK), anti‐pS2808‐RyR2 (1 : 1000; Badrilla), antibodies followed by HRP‐conjugated donkey anti‐rabbit IgG antibodies (GE Health Care). Chemiluminescent detection was performed with WesternBright™ Chemiluminescent Substrate (Biozym Scientific GmbH, Hess. Oldendorff, Germany). Phosphorylation levels of the proteins were normalized to total protein expression.

### Statistical analysis

Data are presented as means ± SEM. Statistical analyses were performed using one‐way ANOVA with Tukey's correction for multiple comparisons. *P* < 0.05 was considered statistically significant. Analyses and graphs were generated using graphpad prism 9 Statistical Software (GraphPad, San Diego, CA, USA).

## Results and Discussion

We hypothesized that CaMKII‐specific RyR2 Ser‐2814 phosphorylation could be the central mechanism by which SR Ca^2+^ leak develops in acute β1‐adrenergic stress. We investigated this hypothesis by exposing cardiomyocytes from a mouse model harboring the phosphoresistant S2814A mutation [12, 14] (vs littermate WT cells) to ISO (100 nmol·L^−1^) as compared to control conditions (NT) and measuring SR Ca^2+^ leak using the gold‐standard method of tetracaine‐sensitive Ca^2+^ shift by Shannon *et al*. [5] using Ca^2+^‐sensitive epifluorescence microscopy (Fluo‐4).

**Fig. 1:**
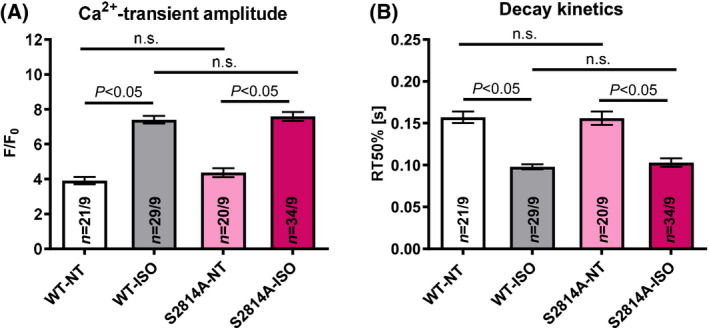
Ca^2+^‐transient amplitude and decay kinetics. Mean data for Ca^2+^‐transient amplitudes (A) and decay kinetics (rt50%) (B) show that both the increase of Ca^2+^ amplitude and the acceleration of Ca^2+^ transient decay are strong in both genotypes upon ISO stress and not different between genotypes. Data are shown as means ± SEM (*n* = cells/mice; one‐way ANOVA).

To assess whether Ser‐2814 phosphorylation might be relevant for the positive inotropic and lusitropic Ca^2+^‐handling responses to adrenergic stimulation, we first compared electrically invoked Ca^2+^ transients in the absence vs. presence of ISO‐stimulation. We can report that both the positive inotropic increase of Ca^2+^ amplitude [WT‐ISO: 7.40 ± 0.22 vs WT‐NT: 3.91 ± 0.21, *n*(WT‐ISO) = 29, *n*(WT‐NT) = 21; *P* < 0.05; S2814A‐ISO: 7.59 ± 0.26 vs S2814A‐NT: 4.37 ± 0.26, *n*(S2814A‐ISO) = 34, *n*(S2814A‐NT) = 20; *P* < 0.05] as well as the positive lusitropic acceleration of Ca^2+^‐transient decay (WT‐ISO: 0.098 ± 0.003 s vs WT‐NT: 0.157 ± 0.007 s, *P* < 0.05; S2814A‐ISO: 0.103 ± 0.005 s vs S2814A‐NT: 0.156 ± 0.008 s, *P* < 0.05; numbers as above) are strong in both genotypes and not different between genotypes (Fig. 1A,B). Thus, neither inotropic (transient amplitude) nor lusitropic (transient decay) response to β1‐adrenergic stimulation depend on RyR2 Ser‐2814 phosphorylation. (And indeed even mice with knockout of the dominant cardiac isoform of CaMKII, CaMKIIδ, show a normal response to adrenergic stimulation [21].)

**Fig. 2:**
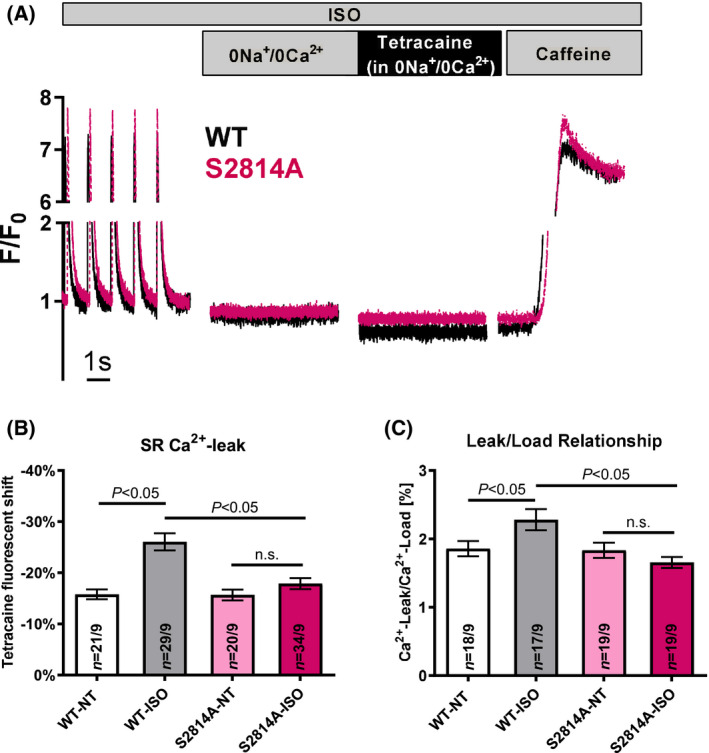
Tetracaine‐sensitive SR Ca^2+^ leak. Original recordings (A) show tetracaine‐sensitive SR Ca^2+^ leak in S2814A and WT mice under ISO stimulation. Mean data (B) show protection from ISO‐induced SR Ca^2+^ leak in phosphoresistant RyR2 S2814A mice as compared to WT mice (*P* = WT‐NT vs. WT‐ISO; *P* = WT‐ISO vs. S2814A‐ISO). Also, ISO‐induced worsening of leak/load relationship (C) was completely prevented in S2814A compared to WT cells. Data are shown as means ± SEM (*n* = cells/mice; one‐way ANOVA).

Importantly, however, we found that cardiomyocytes with the phosphoresistant RyR2 S2814A mutation were completely protected from β1‐adrenergically induced SR Ca^2+^ leak: Under basal conditions (NT), the S2814A mutation had no effect on tetracaine‐sensitive SR Ca^2+^ leak [fluorescence shift upon tetracaine 15.78 ± 0.97% in WT vs. 15.56 ± 1.04% in S2814A; *n*(WT) = 21 cells, *n*(S2814A) = 20 cells; n.s.]. As expected, β1‐adrenergic stimulation with ISO strongly increased the SR Ca^2+^ leak in WT cardiomyocytes [WT‐ISO: fluorescence shift 26.05 ± 1.67%; *n*(WT‐ISO) = 29; *P* < 0.05 vs. WT‐NT]. In S2814A mice, however, ISO stress did not lead to a significant increase of basal SR Ca^2+^ leak [S2814A‐ISO: fluorescence shift 17.88 ± 1.09%; *n*(S2814A‐ISO) = 34; n.s. vs. S2814A‐NT] and these mice were, consequently, protected from ISO‐induced SR Ca^2+^ leak as compared to WT (*P* < 0.05, means/SEM and numbers as above; Fig. 2A,B). To exclude that possible differences in SR Ca^2+^ content between the genotypes were the reason for the protection from ISO‐induced Ca^2+^ leak rather than the RyR2 function itself, we also measured SR Ca^2+^ load in these cells and calculated leak/load relationship. In contrast to WT myocytes, where ISO significantly increased leak/load relationship, [WT‐ISO: 2.28 ± 0.15% vs. WT‐NT: 1.86 ± 0.11%; *n*(WT‐ISO) = 17; *n*(WT‐NT) = 18, *P* < 0.05], implying increased ‘leakiness’ of the RyR2 upon this adrenergic stimulation, S2814A cells were completely protected from this worsening of leak/load relationship under β1‐stress [S2814A‐ISO: 1.65 ± 0.08% vs. S2814A‐NT: 1.83 ± 0.11%; *n*(S2814A‐ISO) = 19; *n*(S2814A‐NT) = 19, n.s. vs. S2814A‐NT, *P* < 0.05 vs. WT‐ISO; Fig. 2C].

**Fig. 3:**
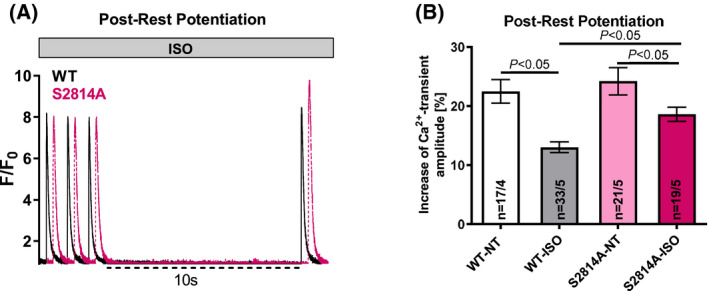
Postrest potentiation of systolic Ca^2+^ release. Original recordings (A) show postrest potentiation of S2814A and WT cells under β‐adrenergic stimulation with ISO. Original recordings from S2814A are depicted time‐shifted vs WT to be visually better discernible. Mean data (B) show that postrest potentiation of systolic Ca^2+^ release was significantly better preserved in S2814A mice under ISO stress compared to WT mice. Data are shown as means ± SEM (*n* = cells/mice; one‐way ANOVA).

Next, we investigated postrest potentiation as an integrative measure for the capacity of the SR to further accumulate Ca^2+^ [22] after 10‐s cessation of stimulation. In WT mice, postrest potentiation was significantly diminished under β1‐adrenergic stress as compared to basal conditions [WT‐ISO: +13.0 ± 0.9% vs. WT‐NT: +22.5 ± 2.0%; *n*(WT‐ISO) = 33, *n*(WT‐NT) = 17; *P* < 0.05], suggesting that the extensively increased SR Ca^2+^ load during ISO stimulation has already reached its maximum. However, while in S2814A mice postrest potentiation was also somewhat lower under β1‐adrenergic stress compared to basal conditions [S2814A‐ISO: +18.6 ± 1.2% vs. S2814A‐NT: +24.2 ± 2.3%; *n*(S2814A‐ISO) = 19, *n*(S2814A‐NT) = 21; *P* < 0.05], it was significantly improved compared to WT cells (*P* < 0.05) (Fig. 3A,B). Thus, phosphorylation of RyR2 at Ser‐2814 during adrenergic stress impairs the ability of the SR to further accumulate Ca^2+^.

**Fig. 4:**
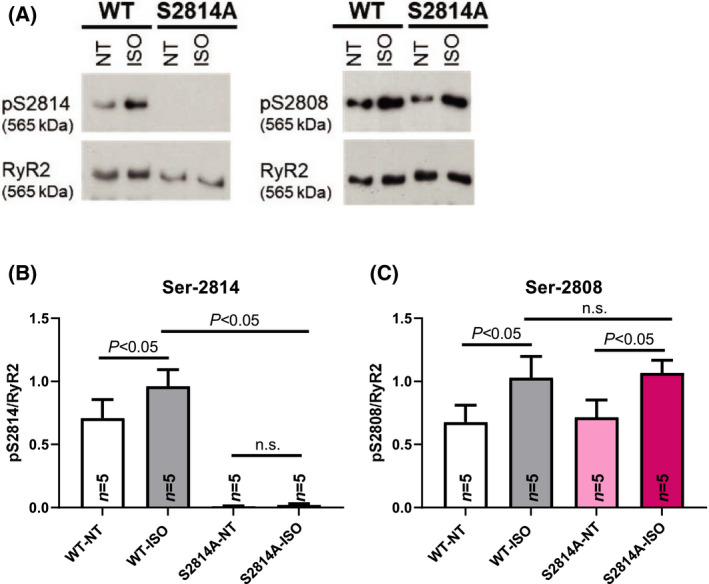
Phosphorylation level of Ser‐2814 and Ser‐2808. Examples of protein expression (A) in WT and S2814A mice with or without ISO stimulation. Mean data of Ser‐2814 (B) and Ser‐2808 (C) phosphorylation levels show phosphoresistance at Ser‐2814 in S2814A mice, while phosphorylation levels at Ser‐2808 were significantly increased upon ISO stress both in WT and S2814A mice. Data are shown as means ± SEM (*n* = mice; one‐way ANOVA).

In order to look into phosphorylation of RyR2 at Ser‐2808 and Ser‐2814 under β‐adrenergic stress and to exclude off‐target effects of the S2814A mutation on Ser‐2808 phosphorylation, we performed western blots in WT and S2814A cells with or without ISO incubation. We can report that the S2814A mutation indeed completely prevents RyR2 Ser‐2814 phosphorylation also under adrenergic stress (Fig. 4A,B). In contrast, phosphorylation of Ser‐2808 is significantly increased by ISO stimulation, but neither the basal phosphorylation (NT) nor the phosphorylation level under adrenergic stimulation at this site are different between WT and S2814A (Fig. 4A,C).

In conclusion, our study defines for the first time phosphorylation of RyR2 at the CaMKII‐specific RyR2 Ser‐2814 phosphorylation site as the pivotal mechanism mediating β1‐adrenergic induced SR Ca^2+^ leak in cardiomyocytes. Phosphoresistance at this site almost prevented ISO‐induced SR Ca^2+^ leak and increase of leak/load relationship. Furthermore, as SR Ca^2+^ leak and leak/load relationship were not different between genotypes under basal conditions, it appears that Ser‐2814 only becomes relevant for SR Ca^2+^ leak upon (phosphorylation) challenge such as under adrenergic stress.

## Conflict of interest

The authors declare no conflict of interest.

## Author contributions

SN conceived and supervised the study. MJB, JN, and MTS designed and performed experiments and analyzed data. MJB, LSM, and SN interpreted data for the work. MJB and JN wrote the manuscript. MTS, LSM, and SN made manuscript revisions.

## Data Availability

The data that support the findings of this study are available from the corresponding author (stefan.neef@ukr.de) upon reasonable request.
